# Integrated in vivo and in silico analysis of immune gene expression in cattle infected with *Brucella abortus*

**DOI:** 10.1038/s41598-025-34173-2

**Published:** 2026-01-17

**Authors:** Dalia M. Mabrouk, Mohamed El-Diasty, Sekena H. Abdel-Aziem

**Affiliations:** 1 Department of Cell Biology, National Research Centre, Giza, 12622 Egypt; 2 Agricultural Research Center (ARC), Animal Health Research Institute-Mansoura provincial Laboratory (AHRI-Mansoura)-Giza, Cairo, 12618 Egypt

**Keywords:** Brucella abortus, Bos taurus, Gene expression, Innate immunity, TLR signaling, Computational biology and bioinformatics, Diseases, Immunology, Microbiology

## Abstract

*Brucella abortus* is an intracellular pathogen that causes infection in cattle, leading to reproductive losses and posing a zoonotic risk to humans. Understanding host immune responses at the molecular level is essential for developing targeted diagnostics and control strategies. This study aimed to investigate the expression of immune-related genes in *Bos taurus* naturally infected with *Brucella abortus* using integrated in vivo and in silico approaches. The infection was characterized as chronic based on persistent seropositivity and historical herd records of infection for 12–18 months prior to sampling. Gene expression analysis revealed significant upregulation of *NOD2* and *IL10*, indicating simultaneous activation of pro-inflammatory signaling and regulatory responses during chronic infection. In contrast, *TLR9* was markedly downregulated, suggesting immune evasion mechanisms that suppress endosomal DNA recognition pathways. The expression levels of *TLR5* and *TLR6* remained unchanged, possibly due to the pathogen’s avoidance of flagellin and lipoprotein recognition. Venn diagram and protein–protein interaction (PPI) analyses highlighted functional overlaps among genes involved in infection response, Toll-like receptor signaling, and KEGG Brucella pathways. GO and KEGG enrichment further confirmed the involvement of the MyD88-dependent TLR signaling pathway, nitric oxide biosynthesis, and pathogen recognition mechanisms. These findings emphasize the complexity of the host immune response to chronic brucellosis, where the immune system attempts to control infection while being subverted by bacterial strategies. The identified gene expression patterns not only enhance our understanding of Brucella pathogenesis but also provide potential molecular markers that could guide future strategies for disease diagnosis and therapeutic intervention. Overall, this study contributes valuable insights into the host-pathogen interactions that define chronic *Brucella abortus* infection in cattle.

**Supplementary Information:**

The online version contains supplementary material available at 10.1038/s41598-025-34173-2.

## Introduction

Brucella abortus, a Gram-negative bacterium capable of intracellular survival, is the causative agent of bovine brucellosis a transmissible disease with significant zoonotic implications for both cattle and human populations globally. Cattle (*Bos taurus*) infected with *B. abortus* primarily experience reproductive disorders, including abortion, placentitis, infertility, and decreased milk production, leading to substantial economic losses in endemic areas^1,2^. Human brucellosis ranks among the most common zoonotic infections, primarily spread via exposure to infected livestock or ingestion of raw dairy products^3^. Despite ongoing control strategies, including vaccination and culling programs, the pathogen’s ability to establish chronic infection and evade immune surveillance poses significant challenges for eradication, especially in developing countries^4^.

Chronic brucellosis in cattle poses unique challenges for diagnosis and research. Infected animals can carry the bacteria for long periods, intermittently shedding it while displaying varying clinical symptoms. This chronic phase involves intricate interactions between the host and pathogen that are not completely understood at the molecular level. While previous studies have looked at immune responses to *Brucella* infection, most have concentrated on acute infection models or laboratory settings, with little examination of naturally occurring chronic infections in real-world conditions^5,6^. Understanding the immune mechanisms during chronic infection is essential for improving diagnostic methods and control strategies.

A defining feature of *Brucella* pathogenesis is its ability to survive and replicate within host macrophages, thereby avoiding extracellular immune mechanisms. Upon entering the host, *Brucella* is engulfed by phagocytes but avoids lysosomal degradation by modulating intracellular trafficking, enabling it to persist in a modified endoplasmic reticulum-derived compartment^7^. Innate immunity heavily depends on pattern recognition receptors (PRRs), such as TLRs and NLRs, which recognize conserved microbial motifs (PAMPs) to initiate proinflammatory cascades^8,9^.

Among the TLR family, TLR9 detects unmethylated CpG motifs in bacterial DNA within endosomes, while TLR5 and TLR6 recognize flagellin and lipoproteins, respectively^10,11^. However, *Brucella* employs strategies to limit exposure of its PAMPs. For example, its lipopolysaccharide (LPS) is structurally modified to reduce TLR4 activation, and its flagellin is either absent or expressed in a form that avoids TLR5 recognition^12,13^. Similarly, *Brucella* can suppress TLR9-mediated signaling, diminishing type I interferon and interleukin production, which are vital for bacterial clearance^14,15^. These adaptations complicate host detection and contribute to the establishment of chronic infection.

In contrast, intracellular receptors such as NOD2 remain critical for sensing cytosolic peptidoglycan fragments, triggering NF-κB and MAPK pathways to elicit inflammatory responses^16,17^. Simultaneously, cytokines like interleukin-10 (IL-10) modulate inflammation by limiting the synthesis of inflammatory cytokines, including tumor necrosis factor-alpha (TNF-α) and interleukin-1 beta (IL-1β). While IL-10 plays an essential role in preventing immunopathology, its overexpression can facilitate immune evasion by intracellular pathogens, including Brucella^18,19^.

The complexity of these interactions underscores the importance of analyzing gene expression patterns during chronic infection. Recent advances in transcriptomics and bioinformatics enable the integration of in vivo expression profiles with in silico pathway enrichment tools, facilitating the identification of key regulatory genes and signaling cascades involved in host-pathogen interactions^20–22^. However, studies of naturally infected chronic brucellosis cases in field conditions remain limited, particularly regarding the characterization of host immune responses with consideration of potential confounding factors.

Given the central role of TLRs (TLR5, TLR6, TLR9) in sensing Brucella PAMPs, NOD2 in cytosolic detection, and IL-10 in immunoregulation, we selected these five genes as key representatives of the innate immune dialogue during persistent infection. This study aimed to investigate the expression of immune-related genes (TLR5, TLR6, TLR9, NOD2, and IL10) in Bos taurus naturally infected with *Brucella abortus* using integrated in vivo and in silico approaches. The goal was to provide insights into host-pathogen interactions during chronic infection. While acknowledging limitations including sample size and potential confounding factors, these results could inform the design of diagnostic markers or immunomodulatory treatments for brucellosis management. Pro-inflammatory cytokines like TNF-α and IL-1β are central to the anti-Brucella response, but this study focused on upstream pattern recognition receptors (TLR5, TLR6, TLR9, NOD2) and the key immunoregulatory cytokine IL10 to elucidate the initial sensing and regulatory mechanisms that may dictate the outcome of chronic infection. The integrated in silico analysis was used not for novel gene discovery but to functionally interpret and contextualize the expression patterns of these pre-selected, hypothesis-driven target genes within established immune pathways.

## Methods

### Sample collection and ethical compliance

Peripheral blood samples [10 mL per animal] were obtained from 40 Holstein dairy cows (aged 3–6 years, Bos taurus) at a dairy farm in El-Sharkia, Egypt. All animals are from the same farm and experience identical environmental conditions. Variables such as temperature, humidity, housing quality, access to water and food, and air quality are consistent, thus eliminating them as potential confounding factors that could affect the results.

Infected group (*n* = 20): Animals displaying chronic brucellosis symptoms (e.g., recurrent abortions, placental retention) and confirmed seropositivity via Rose Bengal test (RBT), Buffered Acidified Plate Antigen Test (BAPAT), and complement fixation test (CFT)^23^. Herd records confirmed that these animals had tested seropositive for brucellosis for a period of 12–18 months prior to sampling, indicating established, chronic infection. Additionally, Brucella abortus was successfully isolated from placental tissue of at least one animal within the same herd, confirming active infection at the herd level and supporting the chronic infection status of the group. We acknowledge the limitation of vaccination history in the present study. Standard farm biosecurity protocols were in place, and infected animals were managed according to national brucellosis control program guidelines to minimize the risk of transmission within the herd.

Control group [*n* = 20]: Asymptomatic cattle with no history of brucellosis and negative serological results for all three diagnostic assays on two consecutive tests three months apart.

All animals were from the same herd and shared the same environment, management practices, and nutritional conditions. The blood was drawn aseptically from the jugular vein without the use of local anesthesia, as this is a standard and minimally invasive procedure that does not require anesthesia under institutional guidelines. Blood was collected into EDTA tubes and transported on ice to the laboratory within 2 h of collection for immediate RNA preservation.

All procedures adhered to institutional ethical guidelines under approved animal care protocols (Ethics Approval Number: 09440325).

### RNA extraction and cDNA synthesis

Total RNA isolation was performed using TRIzol reagent (Invitrogen, USA) according to the manufacturer’s guidelines. To ensure RNA integrity and minimize changes in gene expression during processing, all blood samples were processed immediately upon arrival at the laboratory. RNA quality assessment included spectrophotometric measurement of purity and concentration (NanoDrop, Thermo Fisher Scientific) along with integrity verification through agarose gel electrophoresis. Only samples with A260/A280 ratios between 1.8 and 2.0 and clear 18 S and 28 S ribosomal RNA bands were used for further analysis. To eliminate genomic DNA contamination, samples were treated with DNase I (RNase-free kit, Thermo Fisher). First-strand complementary DNA was synthesized from 1 µg of total RNA using the RevertAid First Strand cDNA Synthesis Kit [Thermo Fisher Scientific], following the recommended protocol.

### Quantitative real-time PCR [qRT-PCR]

Gene expression profiling was conducted using quantitative real-time PCR for five target immune genes (TLR5, TLR6, TLR9, NOD2, and IL10). The primer sequences were adapted from established bovine studies and validated for specificity in *Bos taurus* (Table 1). Each 20 µL reaction contained SYBR Green PCR Master Mix [Applied Biosystems], 10 ng of cDNA template, and 200 nM of each primer pair. Amplification cycles were performed on a QuantStudio™ 3 Real-Time PCR System [Applied Biosystems] under the following optimized conditions: initial denaturation at 95 °C for 10 min, followed by 40 cycles of denaturation (95 °C, 15 s), annealing (60 °C, 20 s), and extension (72 °C, 30 s). A final melting curve analysis step (95 °C for 15 s, 60 °C for 1 min, and 95 °C for 15 s) was included to verify reaction specificity. Relative quantification of gene expression was determined using the 2^^[−ΔΔCt]^ method^24^ with β-actin serving as the endogenous control for normalization.

**Table 1:** Primer sequence.

Gene name	Primer sequence	Accession no.	Product size
IL10	AAAGCCATGAGTGAGTTTGACA TGGATTGGATTTCAGAGGTCTT	*NM_174088.1*	155
NOD2	CTGGCTCCGAGGAAACACTT GTGCTCAGATGTCGTCCCAT	*NM_001002889.1*	158
TLR5	CCTCCTGCTCAGCTTCAACTAT TATCTGACTTCCACCCAGGTCT	*NM_001040501.2*	172
TLR6	AACTTTGTTGCCGGCAAGAG ACTCGCTCTGGACAAAGTTG	*NM_001001159.1*	103
TLR9	AAGGCTTGAGGAACCTGGTC GTTATTGTCCCGGAGACGCA	*NM_183081.1*	119
β-Actin	GCAAATGCTTCTAGGCGGACT CAATCTCATCTCGTTTTCTGCG	*NM_173979.3*	85

### Computational bioinformatics analysis

The integrated in silico analysis was employed not for novel gene discovery but to functionally interpret and contextualize the expression patterns of these pre-selected, hypothesis-driven target genes within established immune pathways.

### Protein interaction network mapping

Molecular interactions were investigated using STRING v11.5 [https://string-db.org] with Bos taurus as the reference genome. The five target genes were analyzed with a high-confidence interaction threshold [score > 0.700], encompassing both functional and physical associations. Network topology was characterized by node-edge visualization, with key parameters including node connectivity, edge density, clustering coefficient, and statistical significance (PPI enrichment p-value < 1.0e-16) automatically computed by the platform.

### Functional annotation analysis

Gene Ontology classification was performed using DAVID Bioinformatics Resources v6.8 and STRING’s integrated GO module. Biological processes with a p-value < 0.05 were considered significant, including immune response pathways, microbial pattern recognition, and TLR signaling cascades. The analysis focused on elucidating gene functions in the context of Brucella-host interactions.

## KEGG pathway enrichment analysis

KEGG pathway analysis was conducted using both STRING and DAVID interfaces. Statistically significant pathways (*p* < 0.05) were identified, particularly those related to bacterial infection responses and inflammatory mechanisms. Special attention was paid to TLR signaling [bta04620], brucellosis-specific pathways (bta05120), and related immune regulation networks.

### Venn diagram analysis

As part of the in-silico evaluation, a Venn diagram analysis was conducted to explore the overlap among three biologically relevant gene sets involved in *Brucella abortus* infection in *Bos taurus*. The purpose of this analysis was to identify genes that are commonly involved in^1^ immune responses observed in infected animals^2^, the Toll-like receptor (TLR) signaling pathway, and^3^ the KEGG Brucellosis pathway.

The first gene set included immune-related genes that showed differential expression in infected cattle based on in vivo qRT-PCR analysis, specifically TLR5, TLR6, TLR9, NOD2, and IL10. The second set, consisting of genes annotated under gene sets associated with Toll-like receptor signaling were extracted from KEGG (Kyoto Encyclopedia of Genes and Genomes), a widely-used resource for pathway annotation and analysis (bta04620). The third set, representing genes involved in the Brucellosis-specific immune response, was also extracted from KEGG under the Brucellosis pathway (*bta05120)*, both filtered for *Bos taurus*.

The comparison of these three gene sets intersection analysis between gene sets was conducted using Venny 2.1, an interactive online visualization platform (https://bioinfogp.cnb.csic.es/tools/venny/). This tool allowed visual representation of shared and unique genes among the datasets in the form of a three-set Venn diagram. The resulting diagram highlighted *TLR5* as the only gene shared between the Infected and TLR Pathway sets, *NOD2* as the common gene between the Infected and KEGG *Brucella* pathways, and *TLR9* as the sole overlapping gene between the TLR Pathway and Brucellosis pathway sets. No gene was found to be common across all three groups.

### Statistical analysis

Data represented as mean values ± standard error. The Statistical Package for Social Sciences (SPSS version 16.0, SPSS Inc., Chicago, IL, USA) was utilized for this analysis. Since the data followed a normal distribution, a Student’s t-test was utilized to compare the infected and control groups. Significance was typically set at *P* < 0.05.

## Results

### mRNA quantification study

**Fig. 1:**
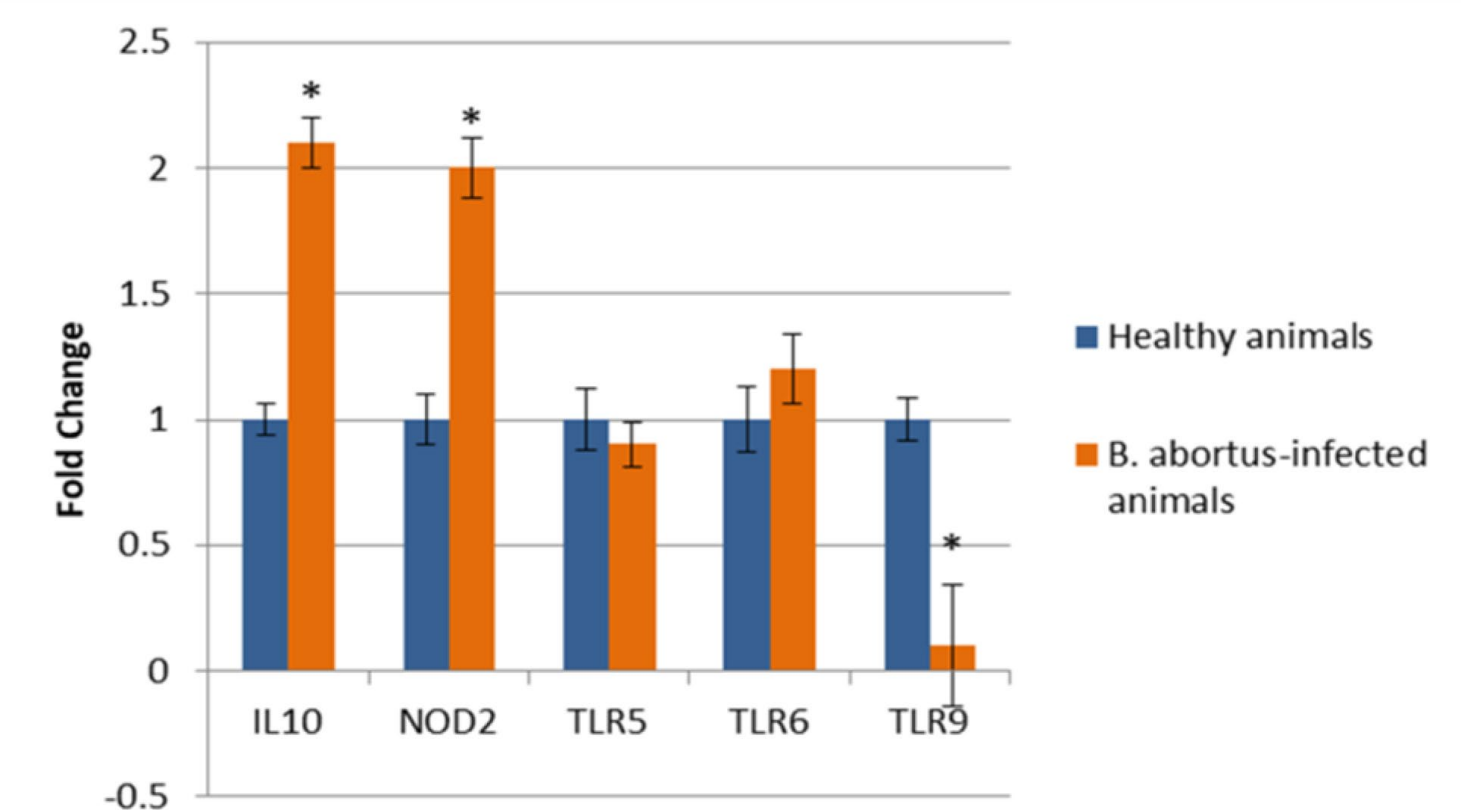
The mRNA expression levels of TLR5, TLR6, TLR9, NOD2, and IL10relative to β-actin gene in animals infected with *B.abortus* (Cases) and Controls. *p < 0.05.

Gene expression analysis in cattle infected with *Brucella abortus* revealed distinct regulation patterns among the immune-related genes examined (Fig. 1). Notably, the NOD2 and IL10 genes exhibited significant upregulation (2.4-fold and 3.1-fold, respectively, *p* < 0.05), indicating an enhanced pro-inflammatory and immunomodulatory response associated with chronic infection. In contrast, the expression of TLR9 was downregulated (2.8-fold, *p* < 0.05), suggesting potential suppression of specific toll-like receptor-mediated recognition of bacterial components. Meanwhile, the expression levels of TLR5 and TLR6 remained unchanged (*p* > 0.05), implying their limited or non-differential involvement in the chronic phase of Brucella infection. These findings underscore the selective activation and suppression of innate immune genes in response to persistent Brucella exposure, reflecting a complex interplay between host defense mechanisms and pathogen evasion strategies. However, we acknowledge that these fold changes, while statistically significant, may have limited biological significance without further validation in larger cohorts (Fig. 1).

## In silico study

### Protein–protein interaction [PPI] network

**Fig. 2:**
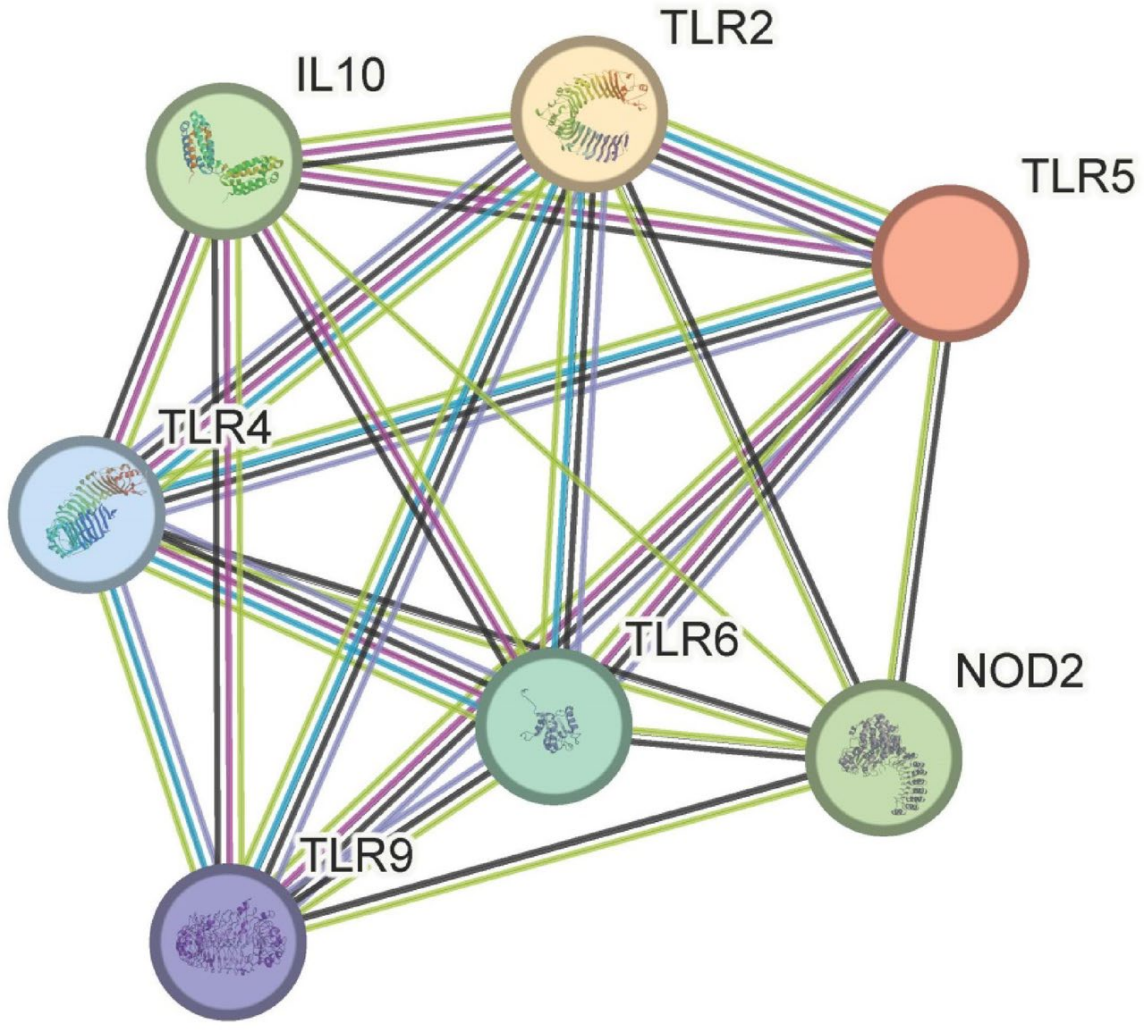
Interaction network of toll-like receptors [TLRs] in *Bos taurus*.

The STRING database analysis of seven immune-related genes in *Bos taurus*, as shown in Fig. 2, revealed a highly interconnected protein–protein interaction (PPI) network. The network consisted of 7 nodes and 21 edges, significantly exceeding the expected edge count of only 1. This suggests that these proteins are functionally associated rather than interacting by chance. The average node degree was 6, indicating that each protein is, on average, connected to six others in the network. Furthermore, the average local clustering coefficient was 1.0, reflecting a tightly clustered group of interacting proteins. The PPI enrichment p-value was **<** 1.0e–16, confirming that the observed interactions are statistically significant and likely biologically meaningful, particularly in the context of the host immune response to *Brucella abortus* infection, as shown in Table 2. These analyses, while insightful for the selected gene set, are based on a limited number of input genes and should be interpreted as a functional contextualization rather than a comprehensive network discovery.

**Table 2:** STRING interaction network Summary.

Metric	Value
Number of nodes	7
Number of edges	21
Expected number of edges	1
Average node degree	6
Average local clustering coefficient	1.0
PPI enrichment p-value	< 1.0e-16

### Gene ontology [GO] biological process enrichment

**Fig. 3:**
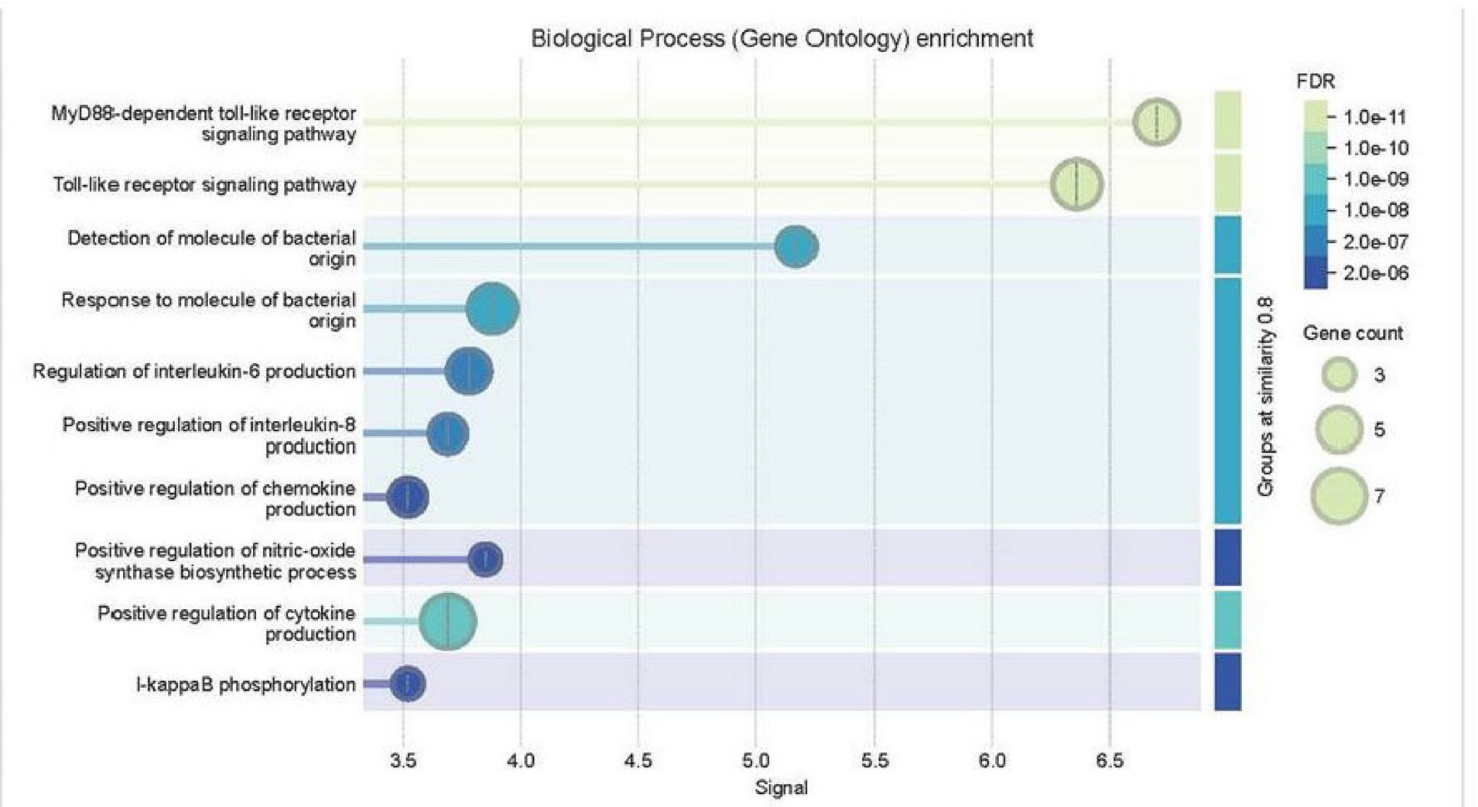
Enriched GO biological processes in immune response to *Brucella abortus* in cattle.

Gene Ontology enrichment analysis focused on biological processes in Fig. 3 revealed several highly significant pathways associated with innate immunity. The most enriched process was the significant activation observed in the MyD88-linked toll-like receptor transduction pathway (GO term 0002755; *p* = 2.01e–11), which plays a central role in initiating immune responses to bacterial pathogens. Other significantly enriched processes included the general toll-like receptor signaling pathway (GO:0002224; *p* = 1.21e–11), as well as the detection and response to molecules of bacterial origin (GO:0032490 and GO:0002237), both of which are critical in recognizing components of invading *Brucella* bacteria. Additionally, the genes showed involvement in the positive regulation of nitric-oxide synthase biosynthetic processes (GO:0051770; *p* = 9.30e–07), highlighting their role in producing reactive molecules during inflammation and bacterial clearance (Table 3).

**Table 3:** Top enriched GO biological Processes.

GO Term ID	Description	*p*-value
GO:0002755	TLR signaling mediated by MyD88 adaptor protein	2.01e-11
GO:0002224	Canonical toll-like receptor signaling cascade	1.21e-11
GO:0032490	Detection molecule of bacterial origin	6.76e-09
GO:0002237	Cellular response to bacterial components	8.09e-09
GO:0051770	Activation of nitric oxide production pathways	9.30e-07

#### K EGG pathway enrichment

Pathway enrichment analysis using the KEGG database identified several significant immune-related signaling pathways involving the selected genes. The most relevant pathway was the Toll-like receptor signaling pathway [bta04620], which plays a key role in recognizing *Brucella* and initiating appropriate immune responses. Additional enriched pathways included those associated with inflammatory diseases and bacterial infections, such **as** inflammatory bowel disease [bta05321], tuberculosis [bta05152], Chagas disease [bta05142], and malaria [bta05144]. These pathways share molecular components and signaling mechanisms with the host response to *Brucella abortus*, suggesting that the identified genes are involved in broadly conserved inflammatory and pathogen recognition mechanisms Table 4.

**Table 4:** KEGG pathway enrichment.

KEGG Pathway ID	Pathway Name
bta04620	Toll-like receptor signaling pathway
bta05321	Inflammatory bowel disease
bta05152	Tuberculosis
bta05142	Chagas disease
bta05144	Malaria

### Venn diagram analysis of genes related to *Brucella* infection in cattle

**Fig. 4:**
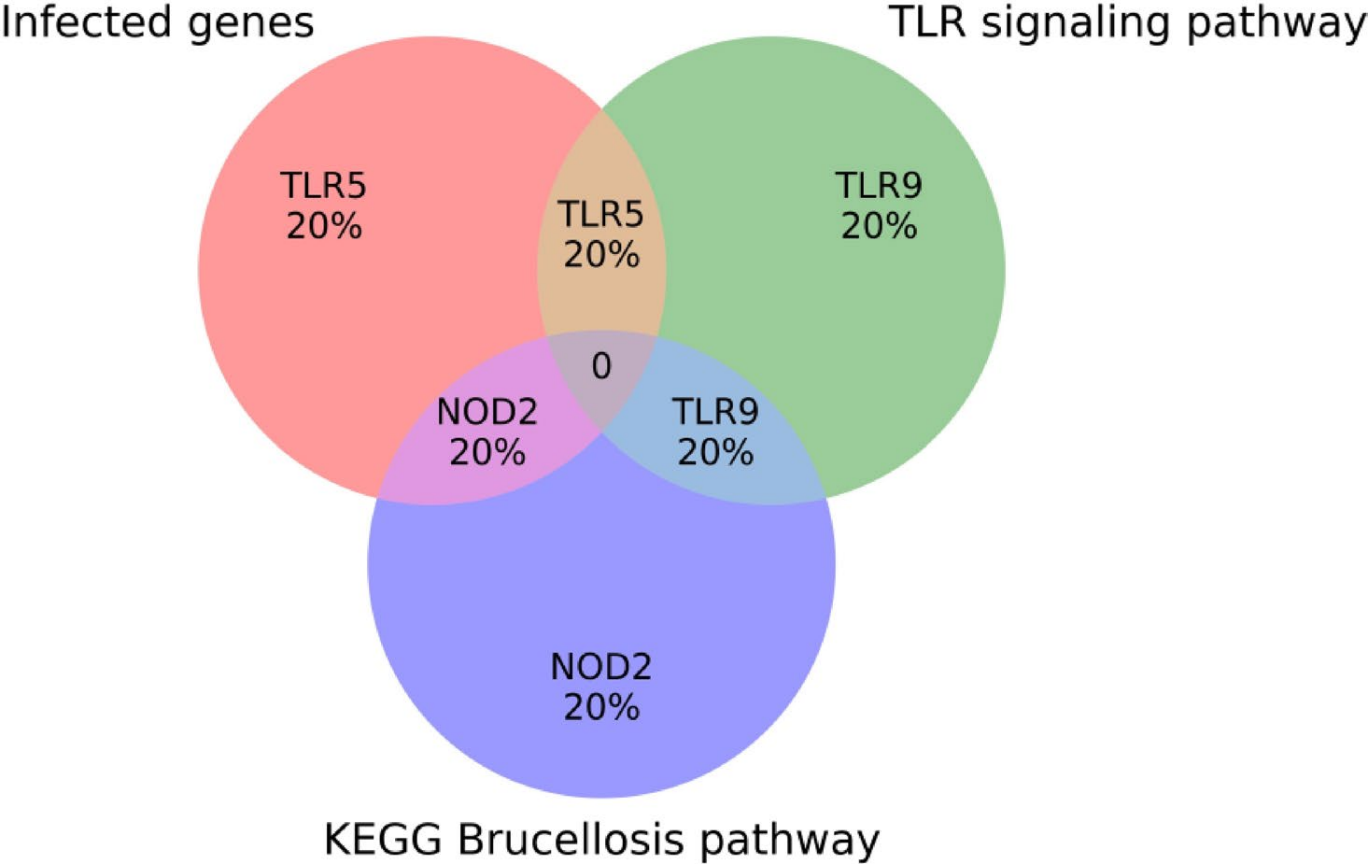
Venn Diagram Showing Overlap between Genes Related to Brucella abortus Infection, TLR Signaling, and KEGG Brucella Pathway in Bos taurus. The diagram shows TLR5 in the intersection between Infected and TLR Pathway sets, NOD2 in the intersection between Infected and KEGG Brucella pathways, and TLR9 in the intersection between TLR Pathway and KEGG Brucella sets.

The Venn diagram analysis compared three gene sets related to the immune response in *Bos taurus* during *Brucella abortus* infection: genes significantly expressed in infected animals, genes involved in the TLR signaling pathway, and genes annotated in the KEGG Brucellosis pathway (Fig. 4).

The analysis revealed that only one gene (TLR5) was shared between the Infected and TLR Pathway sets. Additionally, NOD2 was common between the Infected and KEGG Brucella pathways, while TLR9 was the only gene shared between the TLR Pathway and KEGG Brucella sets. No gene was common to all three groups, indicating distinct yet functionally complementary roles of these genes in the host immune response.

This pattern suggests that while the selected genes are all relevant to Brucella-induced immunity, they contribute through diverse but intersecting signaling mechanisms. It also highlights potential gene candidates that might serve as biomarkers or therapeutic targets specific to the Brucella response, though further validation is needed.

## Discussion

The present study elucidates the transcriptional behavior of critical immune-related genes in *Bos taurus* during *Brucella abortus* infection, offering insights into the molecular underpinnings of persistent host-pathogen interaction. While our findings provide valuable preliminary data, we acknowledge several limitations including the relatively small sample size and potential confounding factors that may influence gene expression patterns in field conditions.

The upregulation of NOD2 and IL10 reflects the host’s attempt to sustain inflammatory defense while simultaneously limiting immunopathology. NOD2 is a well-characterized intracellular sensor of bacterial peptidoglycan that triggers downstream NF-κB and MAPK signaling, crucial for inflammatory cytokine production^16,24,25^. The elevated expression of NOD2 in infected cattle suggests continuous intracellular recognition of Brucella components, which aligns with prior findings indicating that NOD2 plays an essential role in granulomatous inflammation and bacterial containment in Brucella infections^27,28^.

The concomitant upregulation of IL10 may indicate an immunoregulatory feedback mechanism attempting to modulate excessive inflammation. Through its immunosuppressive effects, IL-10 blocks the production of proinflammatory cytokines including TNF-α, IL-1β and IL-6 and limits antigen presentation by dendritic cells and macrophages^17,26^. In the context of chronic infection, this regulatory environment may favor Brucella persistence by suppressing effector functions necessary for bacterial clearance^14,26,29^. This duality may reflect the pathogen’s ability to exploit immune tolerance mechanisms for intracellular survival.

The downregulation of *TLR9*, a DNA-sensing receptor that detects unmethylated CpG motifs in bacterial DNA within endosomes, may highlight a potential immune evasion strategy by *Brucella*. Previous studies have shown that *Brucella* actively suppresses endosomal maturation and avoids TLR9-mediated detection, thereby blunting the type I interferon response and delaying immune activation^30–32^. This suppression may also hinder inflammasome activation, a mechanism typically required for efficient bacterial clearance.

Interestingly, the expression levels of TLR5 and TLR6 remained unchanged. TLR5 is responsible for recognizing bacterial flagellin. However, Brucella abortus either lacks a functional flagellin gene or expresses a modified version that prevents detection by TLR5. This is in line with previous findings indicating that the immune system can be evaded through altering the structure of antigens^33–35^. TLR6, which typically forms a complex with TLR2 to detect lipoproteins, may also be avoided by Brucella. This is likely due to the presence of unique lipoproteins in its outer membrane that do not effectively activate TLR2/6 signaling^35,36^.

Supporting these transcriptional findings, gene ontology analysis may reveal significant enrichment in pathways associated with toll-like receptor signaling and detection of bacterial molecules, especially those involving MyD88-dependent responses. The MyD88 adaptor protein is pivotal in initiating cytokine production in response to TLR activation, and its importance in defense against *Brucella* has been confirmed in knockout models^15,37^. Enrichment in nitric oxide biosynthesis pathways further suggests that the host attempts to utilize reactive nitrogen species for pathogen control an immune mechanism well-documented in *Brucella*-infected macrophages^38,39^.

Pathway enrichment using KEGG also revealed overlap with tuberculosis and inflammatory bowel disease signaling, which share conserved innate immune components such as TLRs, NOD-like receptors, and proinflammatory cytokines. These overlaps emphasize the broad relevance of the identified genes in chronic inflammatory and infectious diseases beyond brucellosis^40^.

The Venn diagram and protein–protein interaction network analysis emphasize that genes like *TLR5*, *TLR9*, and *NOD2* are functionally interconnected, but their regulatory roles are context-dependent^41^. *TLR5* is a common component in both the infected and TLR pathway gene sets, highlighting its conserved involvement in immune signaling, even if its expression remains unchanged^42^. Meanwhile, the KEGG pathway analysis identified overlaps with other bacterial and inflammatory diseases such as tuberculosis and inflammatory bowel disease, reflecting the conserved nature of the immune signaling pathways activated by different pathogens^43,44^. While our findings provide valuable preliminary data from a naturally infected cohort, we acknowledge that the moderate sample size is a limitation, and further studies with larger populations are necessary for validation.

## Conclusions

Collectively, these results not only support existing literature on *Brucella* pathogenesis but also contribute new insights into how persistant infection modulates host immunity through selective gene expression. The identified upregulation of *NOD2* and *IL10* alongside *TLR9* suppression may highlight a dual strategy of immune activation and regulation an interplay that *Brucella* likely exploits to establish long-term persistence within its host. While our study has limitations, including sample size and potential confounding factors, the identified molecular signatures may facilitate the development of novel diagnostic biomarkers or targeted immunotherapies to better control chronic brucellosis in livestock and potentially humans. Future studies with larger cohorts, longitudinal sampling, and more comprehensive transcriptomic profiling are needed to validate and expand upon these findings.

## Supplementary Information

Below is the link to the electronic supplementary material.

Supplementary Material 1

## Data Availability

The datasets generated and/or analyzed during the current study are available from the corresponding author on reasonable request.
