# Supplementary material for: Integrated in vivo and in silico analysis of immune gene expression in cattle infected with Brucella abortus
Source: Sci Rep. 2026 Jan 17;16:2604. doi: 10.1038/s41598-025-34173-2 (PMC12820187; doi:10.1038/s41598-025-34173-2)
Supplement: Supplementary file 1 — Supplementary Material 1 [file 41598_2025_34173_MOESM1_ESM.docx]

Dear Reviewer,,,,

My research doesn’t use any Gels and Blots images

Thanks…..
